# Comparison of Hippocampal Volume in Dementia Subtypes

**DOI:** 10.5402/2013/174524

**Published:** 2012-12-02

**Authors:** Avinash Vijayakumar, Abhishek Vijayakumar

**Affiliations:** ^1^Department of Radiology, Banaras Hindu University, Uttar Pradesh, India; ^2^Department of General Surgery, Victoria Hospital Bangalore Medical College and Research Institute, No. 128 Vijay Doctors Colony, Konanakunte, Bangalore 560062, Karnataka, India

## Abstract

*Aims*. To examine the relationship between different types of dementia and hippocampal volume. *Methods*. Hippocampal volume was measured using FL3D sequence magnetic resonance imaging in 26 Alzheimer's, vascular dementia, mixed dementia, and normal pressure hydrocephalus patients and 15 healthy controls and also hippocampal ratio, analyzed. Minimental scale was used to stratify patients on cognitive function impairments. *Results*. Hippocampal volume and ratio was reduced by 25% in Alzheimer's disease, 21% in mixed dementia, 11% in vascular dementia and 5% in normal pressure hydrocephalus in comparison to control. Also an asymmetrical decrease in volume of left hippocampus was noted. The severity of dementia increased in accordance to decreasing hippocampal volume. *Conclusion*. Measurement in hippocampal volume may facilitate in differentiating different types of dementia and in disease progression. There was a correlation between hippocampal volume and severity of cognitive impairment.

## 1. Introduction

Brain ageing is a universal phenomenon and affects all. Normal ageing can be defined as a normal biologic process of the elderly characterized by relative cerebral atrophy without severe compromise of normal cognitive and motor functions. The ageing brain shows volumetric decrease, usually associated with diffuse or focal white matter signal abnormalities. A clear clinical or pathologic cutoff between physiologic and abnormal ageing of the brain does not exist, however.

Recent developments in MRI hardware and acquisition techniques hold great promise to more sensitively study brain changes in againg. Structural imaging, historically used to exclude an intracerebral lesion as a cause for dementia, is increasingly playing a role in “ruling in” diagnoses. The recent availability of new treatments for dementia, as well as the importance of subtype-specific management, has renewed interest in the use of brain imaging techniques that can assist in the accurate recognition of Alzheimer's disease (AD), vascular dementia (VD), mixed dementia (MD), and normal pressure hydrocephalus (NPH). MRI has been the primary tool to link hippocampal volume loss with AD firmly. There is also growing interest regarding using MRI in conjunction with biochemical marker of AD (tau protein and amyloid Ab) and identifying early dementia MCI (mild cognitive impairment). 

 Various studies have quantified the amount of hippocampal atrophy in old age and dementia but there is a lack of uniformity regarding the result. There is a paucity of studies in our country regarding the hippocampal volume loss in cognitively normal elderly individuals and dementia. In present study effort has been made to quantify the amount of hippocampal volume loss in old age and dementia and study the effect of dementia on brain atrophy. This opens the possibility to study the ageing brain in an epidemiological context and to define new imaging biomarkers for neurodegenerative and cerebrovascular disease at the population level.

## 2. Patients and Methods

### 2.1. Study Population

The present study was carried out in the Department of Radiodiagnosis and Imaging, Sir Sunderlal Hospital, Institute of Medical Sciences, Banaras Hindu University, Varanasi, during the period from July 2010 to July 2012. Patients presenting to the Department of neurology with history of memory loss formed part of study. A detailed history regarding age, sex, duration of symptoms, and comorbid conditions was taken.

A complete blood count, thyroid function test, serum vitamin B12 and folate estimation, and a syphilis test were performed in order to exclude other causes of dementia. Patients with abnormal laboratory results, intracranial mass lesions, history of brain trauma or other neurological disorders, acute stroke within the past 6 months, or who were dependent on alcohol or psychoactive substances were excluded from the study.

Healthy age-matched volunteers with no neurological, psychiatric, or systemic disease were recruited to the control group.

### 2.2. Diagnosis of Dementia

All study participants were evaluated according to the Diagnostic and Statistical Manual of Mental Disorders (DSM-IV) [1]. 

Alzheimer's disease was diagnosed according to the methods described by the National Institute of Neurological and Communicative Disorders and Stroke and the Alzheimer's Disease and Related Disorders Association (NINCDS-ADRDA) [2]. The National Institute of Neurological and Communicative Disorders and Stroke and Association Internationale pour la Recherche et l'Enseignement en Neurosciences (NINDS-AIREN) [3] criteria were used for diagnosis of vascular dementia. A scoring system developed by Kubo et al. [4] for diagnosis of idiopathic normal pressure hydrocephalus (iNPHS) was used as diagnostic criteria for diagnosis of normal pressure hydrocephalus. 

### 2.3. Neuropsychological Assessment

Cognitive function was evaluated in all participants using the Mini Mental State Examination (MMSE) [5]. Since majority of our subjects did not belong to highly educated group a score of 24 was taken as normal. Subjects were stratified according to MMSE score: severe dementia (<9); moderate dementia (10–17); mild dementia (17–24).

### 2.4. MRI Protocol

All participants underwent MRI after assessment for any contraindications. Scanning was performed in the supine position with a 1.5 T imaging systemmagnetom avanto (Version BV-I7A) Siemens Medical System, Erlanger, Germany. The imaging protocol comprised a coronal T1-weighted FLASH (Fast Low Angle Shot) 3D sequence with slice thickness 1.5 mm, repetition time 14 ms, echo time 4.76 ms, flip angle 25°, and matrix 160 × 256. The boundaries of the hippocampus were outlined manually on each slice according to the previously described criteria [6] by a radiologist who was blind to the diagnosis. 

On coronal viewing, the most anterior slice is the slice on which the hippocampus is first visible. This is seen as a notch in the medial border of the temporal horn of the lateral ventricle; this notch is the border between the amygdala and the hippocampus. The structure of the hippocampus is usually visible, with the white matter of the alveus as a border between the hippocampus and the amygdala. After some slices, the amygdala disappears. The uncal apex of the hippocampus is then visible; sometimes the hippocampus appears to be in two parts—if so, both parts were included. Care was taken not to include choroid plexus. This is more homogeneous grey than the hippocampus. Most of the times, a thin white line is visible on top of the hippocampus: this line corresponds to the alveus and fimbria, which function as a border between the hippocampus and the CSF/choroid plexus. When there is no atrophy or only minor atrophy, the hippocampus will border the temporal stem and midbrain. The tail of the caudate nucleus, the optical tract, and the lateral geniculate nucleus as well as the more posterior part of the pulvinar are grey matter structures that were not included. 

When there is atrophy, the hippocampus is mostly surrounded by CSF. In the most posterior slices, the fimbria continues into the fornix. A straight horizontal line is drawn through the fimbria/fornix at the dorsal border (the more grey appearing part) of the hippocampus. The last, most posterior, slice to measure is the first slice on which the total length of the crus of the fornix is seen. Using manual tracing hippocampus was traced on both sides in every section. The sum of tracing on each side gave the total hippocampal area. To get the hippocampal volume (in cm^3^) the area was multiplied by 0.3 (as slice thickness is 1.5 mm and distance between slices is 1.5 mm). Total hippocampal volume was calculated by summing up right and left hippocampal volumes. 

Hippocampal ratio was calculated by dividing total hippocampal volume and transpineal inner table distance. This was done to normalize the values. 

### 2.5. Statistical Analysis

ANOVA with post hoc analysis was used to compare the means over the study groups. Alzheimer's disease formed one group, vascular dementia formed another, and controls formed the last group. Statistical correlation was carried between the groups for the following variable right hippocampal volume, left hippocampal volume, total hippocampal volume, and hippocampal ratio. The level of statistical significance was kept at *P* < 0.05.

## 3. Results

The study population comprised 41 patients 11 with Alzheimer's disease, 10 with vascular dementia, 3 mixed dementia, 2 normal pressure hydrocephalus, and 15 control subjects. There were no significant between-group differences in age, education, or dominant hand. Mean MMSE scores were significantly lower in patients with vascular dementia compared with all other groups (*P* < 0.001). Demographic data, duration of symptoms and MMSE score are shown in Table 1. 

Statistically significant difference was found (*P* value 0.002) when the AD group was compared with controls and 25% volume reduction in AD was noted (Table 2). When hippocampal volume of vascular dementia group was compared with controls no significant statistical difference was found (“*P*” value being 0.124). But average hippocampal volume of vascular dementia was 11% less than controls. Mixed dementia and NPH showed an average hippocampal volume reduction of 21% and 5% compared to controls though not statistically significant. Similarly statistical significance was found (*P* value < 0.001) for right versus left hippocampal volume and hippocampal ratio, when AD cases were compared with controls. 

 Also no statistically significant reduction in hippocampal volume was demonstrated between AD and vascular dementia (“*P*” value being 0.348). 

Hippocampal volume was reduced by 5% in subjects with mild dementia (*n* = 12), 12% in those with moderate dementia (*n* = 10), and 34% in those with severe dementia (*n* = 4) compared with subjects with normal cognitive function (*n* = 15). The severity of dementia increased in line with decreasing hippocampal volume.

## 4. Discussion 

This study examined the relationship between hippocampal volume and cognitive impairment in patients with Alzheimer's disease, vascular dementia, mixed dementia, normal pressure hydrocephalus, and control subjects, using an MRI-based volumetric method. Mean age of AD group was 71.18 years, mean age of vascular dementia group was 62.20 years, and that in controls was 66.27 years. In a related study by Schmidt [7] mean age of patients probable Alzheimer's disease was 68.2 years, as against mean age of 69.9 years for vascular dementia, and 66.3 years for normal controls. We found that 42.30% cases were in AD group, 11.53% were in mixed dementia group, 38.4% were in vascular dementia group, and 7.60% were in NPH group. According to Knopman et al. [8] pathological diagnoses were AD 51%, pure VD in 13%, mixed dementia in 12%, and other diagnoses in the remaining patients. In another study by Schmidt [7], 53.4% were probable AD, and 46.6% were patients of vascular dementia. In a study by Nair et al. [9] among those with dementia, 45.7% had AD (probable or possible), 22% had VD (probable or possible), 15% had mixed dementia, and the rest had dementia due to other causes.

Severity of dementia in the present study was assessed by MMSE/HMSE score. Average MMSE/HMSE score of AD cases in our study was 18.18, average MMSE/HMSE score of vascular dementia was 12.4, average MMSE/HMSE score of mixed dementia was 13, and average score of NPH cases was 22.50. In a study by Nair et al. [9] average MMSE/HMSE score in AD cases was 17.12 and vascular dementia cases was 16.30. Average MMSE/HMSE scores of the present study correlate well with MMSE/HMSE scores of other studies in our subcontinent. Average MMSE/HMSE score of present study in demented group was 19.29, in a study by Frisoni et al. [10]; mean MMSE score was 18.85.

Our study showed an average of total hippocampal volume of cases in AD group was 3.916 (±0.3) cm^3^, in vascular dementia group was 4.660 (±0.22) cm^3^, and in controls was 5.202 (±0.76)  cm^3^ (Table 2).

Statistically significant difference was found (*P* value 0.002) when AD group was compared with controls and 25% volume reduction in AD was noted. In a study by Frisoni et al. [11] a 32.5% volume reduction in total hippocampal volume was noted (average age 76 ± 6 years, Average MMSE 13 ± 4) in AD as compared to control subjects.

 Giedd et al. [12] studied the hippocampal volume (normalized relative to the size of the lenticular nucleus) and has concluded hippocampal volume to be reduced by 40% in the AD group compared to the controls. In a study by Du et al. [13] total hippocampal volume of patients with NC (normal cognition) was 6327 mm^3^ and 4595 mm^3^ in those with AD, with 27% volume reduction in AD cases. Slightly higher volumes noted in this study could be due to a difference in ethnicity of cases and controls and difference in boundaries of hippocampus (boundaries of the hippocampus were drawn including the hippocampus proper, dentate gyrus, subiculum, fimbria, and alveus.).

When hippocampal volume of vascular dementia group was compared with controls no significant statistical difference was found (“*P*” value being 0.124) though average hippocampal volume of vascular dementia cases was 11% less than average hippocampal volume of control group. In a study by Du et al. [14] when comparison of hippocampal volume between cognitively normal aged controls and patients of SIVD (subcortical vascular dementia) was done, 18.2% smaller hippocampal volume was demonstrated in vascular dementia with statistically significant *P* value. 

Also no statistically significant reduction in hippocampal volume was demonstrated between AD and vascular dementia (“*P*” value being 0.348). But AD patients showed a 17.5% reduction in hippocampus volume compared to VD patients. Gosche et al. [15] have also failed to demonstrate statistically significant difference in hippocampal volume between vascular dementia and AD. Scher et al. [16] studied changes in hippocampal volume in patients of vascular dementia and AD and concluded that hippocampal volume was about 5% smaller in the Alzheimer's disease group compared to the VD group, and the difference was not significant. This would mean that a percentage of reduction of hippocampal volume can be used as a correlative parameter but not as an objective one while trying to subclassify dementia based on hippocampal volume.

Similarly statistical significance was found (*P* value < 0.001) for right, left hippocampal volume (Table 2), and hippocampal ratio when AD cases were compared with controls. Average hippocampal volume in AD in our study was 1.921 cm^3^, average volume of hippocampus in control was 2.60 cm^3^ and vascular dementia 2.299 cm^3^. In a study by Schuff et al. [17] hippocampal volume in control averaged 2133 (±25) mm^3^, patients with mild cognitive impairment averaged 1846  (±23) mm^3^, and clinical AD averaged 1631 (±34) mm^3^.

Average left hippocampal volume in AD, in our study was 1.846 (±0.21) cm^3^ and right hippocampal volume in AD cases was 1.997 (±0.25) cm^3^. Average left hippocampal volume in vascular dementia was 2.370 cm^3^ (±0.35) cm^3^ and right hippocampal volume in vascular dementia cases was 2.29 (±0.33) cm^3^. Many studies have repeatedly demonstrated asymmetric volume reduction in AD with left hippocampus being smaller than right in AD. According to Carne et al. [18] this however reflects the physiological hemispheric asymmetry of the human hippocampus which has been consistently demonstrated in a large number of studies involving healthy subjects of all ages but Müller et al. [19] noted left hippocampus may be more vulnerable in AD than right hippocampus due to smaller volume in the left.

 In a longitudinal study over a three-year period Fox et al. [20] found significantly smaller initial left hippocampi in those subjects who became demented during the observation period and concluded that hippocampal asymmetry may be an early sign of the presence of a degenerative process. Laakso et al. [21] also found smaller left hippocampal volumes in AD compared to Parkinson's disease and vascular dementia. In relation to higher discriminative power of the left hippocampus, Krasuski et al. [22] reported that left-hemispheric volumetric measures of medial temporal lobe structures provided a higher accuracy of group discrimination in a sample of mildly demented AD patients versus controls. 

There were differences in hippocampal volume between patients with moderate or severe dementia and subjects with normal cognitive function in the present study. Analysis of the pooled study population revealed a relationship between the degree of cognitive impairment and hippocampal atrophy, such that subjects with greater cognitive impairment (low MMSE score) had smaller hippocampal volumes.

## 5. Limitations and Conclusion 

This study had several limitations. Patients were not examined throughout their disease course. There are likely to be differences between the early and late stages of dementia in the same patient and hippocampal atrophy in the late stage of one dementia subtype may be indistinguishable from that in the early stage of another dementia subtype. The diagnosis of dementia subtype may be difficult with a single measurement, and repeated volumetric analyses may have higher discriminatory value. The small size of the study cohort meant that it was not possible to analyze the relationship between severity of dementia and hippocampal volume for each dementia subtype. Further investigation with a larger cohort is needed to examine this relationship.

MR imaging is a rapidly evolving technique with a vast potential application in neuroimaging. Excellent anatomical delineation of hippocampus and cortical structure have made hippocampal volumetry and linear measurement accurate. Manual tracing for hippocampal volumetry is considered gold standard method. Measurement of hippocampal volume may help in differentiating between dementia subtypes and normal ageing. Maximal hippocampal atrophy was in Alzheimers disease followed by vascular dementia and NPH. Serial measurement of hippocampal volume may help in predicting the progression of disease and initiating early treatment. Though MR imaging provides excellent opportunity to depict the anatomical changes in brain ageing process, recent advances in neuroimaging like DWI, MR spectroscopy, DTI, and fMRI need to be conjured to depict the biological changes in brain ageing.

## Figures and Tables

**Table 1:** Demographic characteristics and MMSE scores of subjects.

	AD	Mixed	Vascular	NPH	Control
Age in years	61.83 ± 6.3	64 ± 5.4	62.20 ± 6.8	76.50 ± 4.8	66.27 ± 6.1
Male/Female	7/4	2/1	7/3	1/1	9/6
Symptoms duration in years	1.74 ± 0.6	3.83 ± 0.9	3.13 ± 2.5	0.50	—
MMSE	18.18 ± 2.1	13 ± 1.9	12.40 ± 2.7	22.50 ± 1.0	26.2 ± 1.3

**Table 2:** Hippocampal volume and hippocampal ratio (HR) in subjects.

	Hippocampal volume (in cm^3^)	Hippocampal volume (Left in cm^3^)	Hippocampal volume (right in cm^3^)	HR
Controls	5.202 (±0.76)	2.540 (±0.38)	2.660 (±0.43)	0.420
AD	3.853 (±0.20)	1.846 (±0.21)	1.997 (±0.25)	0.289
Mixed	4.150	1.865	2.225	0.316
Vascular dementia	4.660 (±0.22)	2.370 (±0.35)	2.29 (±0.33)	0.354
NPH	4.941	2.394	2.547	0.360
